# Effects of integrated blood purification on haemodynamics and oxygen metabolism in children with severe sepsis

**DOI:** 10.3389/fmed.2024.1400154

**Published:** 2024-11-05

**Authors:** Hekai Ma, Zhiyuan Wang, Jiahao Geng, Junlin Zhao, Tuanjie Wang, Ling Liu, Yuping Xu, Weiqing Liu, Min Wang, Lan Zhao, Shujun Li

**Affiliations:** Department of Pediatrics, The First Affiliated Hospital of Xinxiang Medical University, Weihui, Henan, China

**Keywords:** blood purification, severe sepsis, haemodynamics, oxygen metabolism, children

## Abstract

**Objective:**

To investigate the effects of integrated blood purification on haemodynamics and oxygen metabolism in children with severe sepsis.

**Methods:**

Clinical data of 12 children with severe sepsis admitted to the pediatric intensive care unit of our hospital between October 2021 and June 2022 were retrospectively analyzed. All patients were treated with integrated blood purification, and changes in haemodynamic parameters, including heart rate, blood pressure, mean arterial pressure and cardiac output, and oxygen metabolism parameters (blood lactic acid, oxygen delivery, oxygen consumption and oxygen extraction rate) were observed before and after treatment.

**Results:**

The heart rate (134[106,160] vs 111[101,128], *p* = 0.037), central venous pressure (9[7,10] vs 8[7,9], *p* = 0.04), stroke output (28[18,43] vs 21[15,31], *p* = 0.01), blood lactate (3.3[2,4] vs 2.5[1.3,3.6], *p* = 0.015), oxygen consumption (165.99[121.44,230.31] vs 124.18[82.51,162.86], *p* = 0.041) and oxygen extraction rate (38.83[31.87,44.62] vs 28.67[21.05,32.72], *p* = 0.019) were decreased, whereas systolic blood pressure (97[83,104] vs 107[94,116], *p* = 0.033) and central venous oxygen pressure (32[29, 37] vs 39[34,46], *p* = 0.005) were increased in the children after treatment compared with before treatment. There were no statistically significant differences in diastolic blood pressure, mean arterial pressure, cardiac output, arterial oxygen pressure and oxygen delivery before and after treatment (all *p* > 0.05).

**Conclusion:**

Integrated blood purification can improve haemodynamic and oxygen metabolism parameters in children with severe sepsis, with a high value in clinical application.

## 1 Introduction

The mortality rate of severe sepsis and septic shock in children remains high. More than 11 million people die from sepsis every year despite the fact that the treatment efficiency for the condition has been significantly improved with the advancement of medical technologies (1). Sepsis affects all age groups, with a death rate of over 20%. In 2017, almost half of all cases of sepsis worldwide involved children, with an estimated 20 million cases involving children under 5 years old. Moreover, 10% of children with sepsis progress to severe sepsis or septic shock, with a high mortality rate (2–4). Sepsis may progress to septic shock if left undetected and not treated promptly, and may lead to uncontrolled vascular tension and even vasoplegic shock, causing haemodynamic and oxygen metabolism disorders in the body, ultimately leading to multiple organ failure and even death (5–11).

Progress has been made in the management of children with severe sepsis, including early identification, early quantitative resuscitation, ventilator-assisted breathing, timely and appropriate administration of antibiotics and control of the source of infection. However, the mortality rate of affected children remains high even when blood purification treatment is provided (12). Single-mode blood purification refers to the application of one blood purification method, whereas integrated blood purification mode refers to the combined use of two or more methods. These methods include the following blood purification techniques (13): continuous veno-venous hemofiltration (CVVH), hemoperfusion (HP), continuous veno-venous hemodiafiltration (CVVHDF), plasma exchange (PE), double plasma molecular adsorption system (DPMAS) and double filtration plasmapheresis (DFPP). The combination of different modes of blood purification is also considered to be a potentially effective treatment method for early septic shock (14).

Integrated blood purification has been widely used in the treatment of severe diseases such as drug and poison intoxication, acute and chronic liver and kidney failure, respiratory failure and severe sepsis, with favorable efficacies achieved (15). However, due to the physiological characteristics of children, such as thin blood vessels and low total extracorporeal circulation blood volume, as well as the limitations of blood purification devices and consumables, the application of integrated blood purification in pediatric patients is limited compared with that in adults, and relevant studies have been rarely reported. Changes in haemodynamic and oxygen metabolism parameters are closely related to the severity and prognosis of sepsis. On this basis, the present study investigates the effects of integrated blood purification on haemodynamics and oxygen metabolism in children with severe sepsis.

## 2 Materials and methods

### 2.1 Study participants

In this retrospective study, 15 children with severe sepsis who received integrated blood purification treatment in the pediatric intensive care unit of our hospital between October 2021 and June 2022 were selected. Among them, 3 were excluded, including 1 patient who died on the day of admission, 1 with nephrotic syndrome and chronic renal insufficiency and 1 with incomplete clinical data, meaning 12 patients were enrolled in the final analysis. The baseline data of these patients are shown in Table 1. There were 6 boys and 6 girls, with an average age of 5.4 years old (range: 2 months–13 years). The causes of severe sepsis included pulmonary infection (6 patients), acute suppurative appendicitis (1), acute diffuse peritonitis (1), skin infection (1), blood infection (2) and hemophagocytic syndrome (1). The inclusion criteria included patients who (1) met the diagnostic criteria for severe sepsis (16), (2) were aged 29 days–17 years old and (3) were treated with two or more blood purification modes. The exclusion criteria included patients who (1) were immunocompromised, (2) died on the day of admission, (3) had inherited metabolic diseases and (4) had incomplete clinical data. This study was reviewed and approved by the ethics committee of the hospital, and signed informed consent forms were obtained from the families of the children prior to the study.

**TABLE 1:** Baseline data of children treated with continuous blood purification.

Patients	Age (yrs)	Sex	Body weight(kg)	Infection sites	Pathogens	Dead	Blood purification mode	PRISM III
Case 1	4.7	M	16	Lung	MARS,EBV	No	CVVH,HP,PE,DPMAS	16
Case 2	12	M	44	Lung	MARS,IFV-B	No	CVVH,CVVHDF	12
Case 3	13	M	36	Lung	MARS,IFV-B	Yes	CVVH,CVVHDF,PE	8
Case 4	12	F	55	Brain and lung	HHV-1,Sau	No	CVVH,CVVHDF	8
Case 5	4.1	F	13	Lung	MARS	No	CVVH,PE	35
Case 6	0.17	F	0.22	Blood	Not detected	Yes	CVVH,PE	27
Case 7	0.9	M	9	Blood	S.m	No	CVVH,PE	3
Case 8	2	M	19	Appendix	S.a	No	CVVH,HP	7
Case 9	1.5	F	10.5	Brain and lung	HHV-1,HPIV-3	No	CVVH,HP	10
Case 10	11	M	46	Brain	S.p	Yes	CVVHDF,DFPP	25
Case 11	0.7	F	8	Brain and blood	HHV-1,TTV	No	CVVH,PE	10
Case 12	3	F	18	Blood	Not detected	No	CVVH,HP	10

MARS, methicillin-resistant *S. aureus*; EBV, Epstein-Barr Virus; IFV-B, influenza B virus; HHV-1, Human Herpesvirus 1; S.m, *Staphylococcus mitis*; S.a, *Staphylococcus aureus*; HPIV-3, Human Parainfluenza Virus type 3; S.p, *Streptococcus pneumoniae*; TTV, torque teno virus; CVVH, continuous veno-venous hemofiltration; HP, hemoperfusion; CVVHDF, continuous veno-venous hemodiafiltration; PE, plasma exchange; DPMAS, double plasma molecular adsorption system; DFPP, double filtration plasmapheresis.

### 2.2 Treatments

All patients were treated according to the latest guidelines for sepsis treatment (5). The treatments included anti-infection, anti-shock, source control, mechanical ventilation and vasoactive drug treatment, with integrated blood purification therapy provided at the same time. During the study period, all patients included in our analysis were under mechanical ventilation as part of the standard intensive care for severe sepsis. This approach was necessary to maintain adequate oxygenation and ventilation, which are critical in the management of these patients.

Integrated blood purification therapy refers to the combination of two or more of the following blood purification techniques: CVVH, HP, CVVHDF, PE, DPMAS and DFPP. The selection of blood purification techniques, which included CVVH, HP and PE, was not arbitrary but carefully tailored to the specific clinical needs of each patient. Decisions on the combination of methods were based on individual factors, such as the patient’s haemodynamic stability, severity of inflammation and overall organ function. This personalized approach aligns with clinical practice, ensuring that each patient receives the most appropriate and effective treatment.

The femoral vein or right jugular vein was selected as the vascular access for blood purification, and the catheterisation was guided by ultrasound. Anticoagulation was used in the extracorporeal blood circuit with 4% citric acid (200 ml:8 g) at a pump speed of 1.2–1.5 × blood flow rate, and 10% calcium gluconate at a speed of approximately 6.1% of that of the anticoagulant. The blood flow rate was 3–5 ml/kg⋅min, the dialysate flow rate was 30 ml/kg⋅h, the replacement fluid rate was 30–45 ml/kg⋅h and the ultrafiltration rate was 3–5 ml/kg⋅h (depending on circulation and fluid load). The predilution mode was selected for replacement fluid administration, and the treatment duration was at least 72 h. The blood purification machine operated for a minimum of 16 h per day for 3 consecutive days, the blood perfusion time was 1–2 h and the plasma exchange time was around 2 h. The extracorporeal circulation blood volume was controlled to be less than 10% of the total circulating blood volume of the patient. The models of the blood purification machines, tubes and filters are shown in Table 2.

**TABLE 2:** The major equipment, instruments, and consumables used in this study.

Conventional ventilator		Maquet critical care AB
Blood purification machine	Fresenius bedside blood purifier	Fresenius Group, Germany
Tubing system for blood purification	Disposable extracorporeal circulation tube	Japan Lifeline Co., Ltd.
	Tubing set for continuous blood purification	Fresenius Group, Germany
Blood filters	Nipro blood filter	HuaShengJunKang (Beijing) Tech & Trade Co., Ltd, China
	Hollow-fiber hemodialyzer	Fresenius Group, Germany
Blood perfusion device	Disposable blood perfusion device	Jafron Biomedical Co., Ltd., China
Plasma exchange device	Plasma separator	Bellco, Italy
Double plasma exchange device	Plasma component separator	AsahiKASEI, Japan
Double plasma molecular adsorption system	Disposable plasma bilirubin adsorber	Jafron Biomedical Co., Ltd., China
Bedside ultrasound machine	Mindray Crius ME7P	Shenzhen Mindray Bio-Medical Electronics Co. Ltd, China
Central venous catheterization kit	Disposable central venous catheter set (7F, 11.5F)	Haolang Technology (Foshan) Limited Co., China
	Disposable central venous catheter set (5F)	Shenzhen Yixinda Medical New Technology Co., Ltd., China
	Disposable central venous catheter kit (16G)	Henan Tuoren MEDICAL Device Co., Ltd., China

### 2.3 Parameters monitored

Arterial and mixed venous blood samples were collected from all pediatric patients for blood routine and blood gas analysis, including hemoglobin concentration (Hb), saturation of arterial blood oxygen (SaO_2_), saturation of venous blood oxygen (SvO_2_), arterial oxygen pressure (PO_2_) and venous oxygen pressure (PvO_2_). A bedside ultrasound examination was performed to obtain cardiac function parameters. Cardiac index (CI) was calculated based on stroke volume (SV), whereas oxygen delivery (DO_2_), oxygen consumption (VO_2_) and oxygen extraction rate (O_2_ER) were calculated using the FICK formulas:

DO_2_ (mL/[min⋅m^2^])=CI (mL/[min⋅m^2^]) × CaO_2_(mL/100 mL) × 10; VO_2_ (mL/[min⋅m^2^])=CI (mL/[min⋅m^2^]) × (CaO_2_-CvO_2_)(mL/100 mL) × 10; CaO_2_=Hb × 1.34 × SaO_2_+0.003 × PO_2_; CvO_2_=Hb × 1.34 × SvO_2_+0.003 × PvO_2_; O_2_ER=VO_2_/DO_2_

### 2.4 Statistical analysis

Data were analyzed using SPSS 26.0 software. Measurement data with a normal distribution were presented as mean ± standard deviation, and those without a normal distribution were presented as median and interquartile range. A two-correlated-sample nonparametric rank sum test was used to analyze the data before and after treatment. Given the small sample size and the non-normal distribution of the data, nonparametric tests were selected as they are more appropriate for handling skewed data and outliers. Differences with a *p*-value of <0.05 were considered statistically significant.

## 3 Results

### 3.1 Haemodynamic monitoring results

**FIGURE 1:**
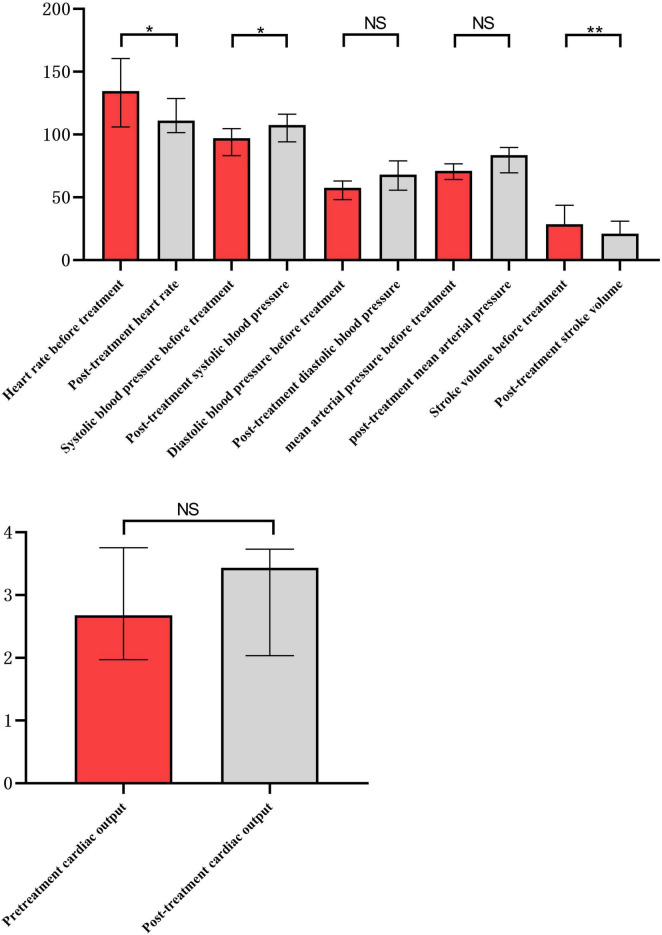
Comparison of hemodynamics before and after integrated blood purification treatment. *Indicates *P* < 0.05, and **indicates *P* <0.01.

There were statistically significant differences in heart rate, systolic blood pressure, central venous pressure and stroke output before and after integrated blood purification treatment (*p* < 0.05), whereas no statistically significant differences were observed in diastolic blood pressure, mean arterial pressure and cardiac output before and after treatment (Figure 1 and Table 3).

**TABLE 3:** Hemodynamic parameters of patients before and after integrated blood purification treatment.

Groups variables	Before treatment (*n* = 12)	After treatment (*n* = 12)	*Z*-value	*P*-value
Heart rate (/min)	134 (106, 160)	111 (101, 128)	−2.090	0.037
Systolic pressure (mmHg)	97 (83, 104)	107 (94, 116)	−2.136	0.033
Diastolic pressure (mmHg)	57 (48, 63)	68 (55, 79)	−1.883	0.06
Mean arterial pressure (mmHg)	71 (64, 76)	83 (69, 89)	−1.806	0.071
Central venous pressure (mmHg)	9 (7, 10)	8 (7, 9)	−2.049	0.04
Stroke volume (ml)	28 (18, 43)	21 (15, 31)	−2.581	0.01
Cardiac output (L/min)	2.68 (1.97, 3.75)	3.43 (2.03, 3.73)	−0.510	0.61

### 3.2 Oxygen metabolism monitoring results

**FIGURE 2:**
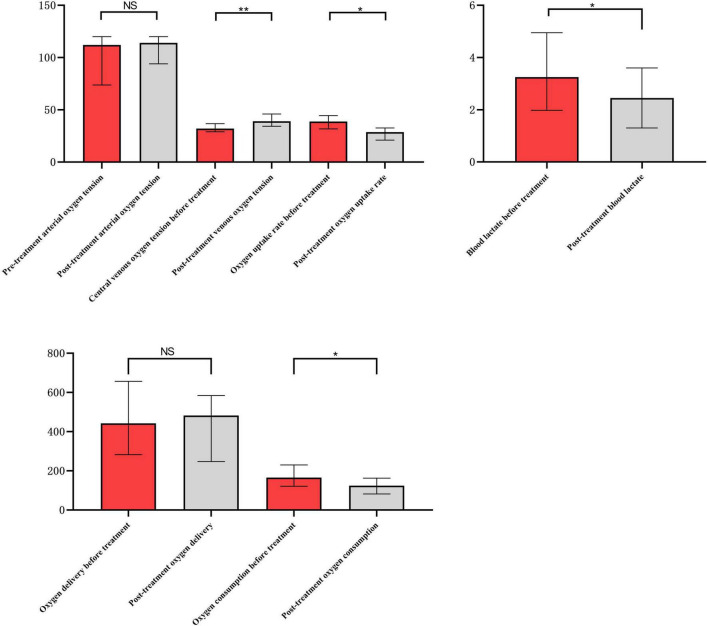
Comparison of oxygen metabolism before and after integrated blood purification treatment. *Indicates *P* < 0.05, and **indicates *P* < 0.01.

There were statistically significant differences in blood lactate level, central venous oxygen pressure, oxygen consumption and oxygen extraction rate before and after integrated blood purification treatment (*p* < 0.05), whereas no statistically significant differences were observed in arterial oxygen pressure and oxygen delivery before and after treatment (Figure 2 and Table 4).

**TABLE 4:** Oxygen metabolism parameters of patients before and after integrated blood purification treatment.

Groups variables	Before treatment (*n* = 12)	After treatment (*n* = 12)	*Z*-value	*P*-value
Blood lactate (mmol/L)	3.3 (2.0, 4.0)	2.5 (1.3, 3.6)	−2.432	0.015
Arterial oxygen pressure (mmHg)	112 (74, 120)	114 (94, 120)	−0.946	0.344
Venous oxygen pressure (mmHg)	32 (29, 37)	39 (34, 46)	−2.808	0.005
Oxygen delivery (ml/min⋅m^2^)	442.62 (282.74, 657.15)	482.41 (247.78, 584.60)	−0.314	0.754
Oxygen consumption (ml/min⋅m^2^)	165.99 (121.44, 230.31)	124.18 (82.51, 162.86)	−2.040	0.041
Oxygen extraction rate (%)	38.83 (31.87, 44.62)	28.67 (21.05, 32.72)	−2.353	0.019

## 4 Discussion

The findings of this study demonstrate that integrated blood purification significantly improves haemodynamic stability and oxygen metabolism in children with severe sepsis. These improvements have direct clinical applications, as they suggest that integrated blood purification can be an effective adjunctive therapy for stabilizing pediatric patients with severe sepsis.

Clinically, the use of integrated blood purification allows for the continuous removal of inflammatory mediators, regulation of fluid balance and enhancement of cardiovascular function, which are crucial in managing septic children. For instance, improvements in heart rate, systolic blood pressure and stroke output observed in this study indicate better cardiovascular support and reduced cardiac workload, which can help prevent further deterioration in critically ill patients. Blood purification can regulate fluid balance and remove harmful metabolites through dispersion, convection and adsorption to reduce blood volume load and improve tissue perfusion, which further improves the haemodynamic status of patients. A previous study (17) found that continuous blood purification (CBP) treatment for severe sepsis in children can improve cardiovascular function, inflammatory indicators and tissue perfusion, and can reduce the mortality of these children.

When both oxygen delivery and oxygen extraction rate are not sufficient to meet the oxygen demand in the tissue, cells experience hypoxia, leading to the incidence of multiple organ dysfunction syndrome (11). He et al. (18) investigated the changes in clinical indicators in patients with severe infections treated with integrated blood purification and found that indicators of the liver and kidney function, inflammation and oxygen metabolism were effectively improved in these patients, with a better oxygen metabolism status.

The results of the present study indicated that the blood lactate level of patients was significantly improved after integrated blood purification treatment. Moreover, we observed reductions in blood lactate levels and oxygen extraction rates, highlighting enhanced oxygen utilization and metabolic efficiency, which are critical in reversing tissue hypoxia and preventing multiple organ dysfunction. These findings suggest that integrated blood purification not only supports haemodynamic parameters but also plays a vital role in optimizing cellular oxygen use, which is essential in severe sepsis management. Rossetti et al. (19) assessed the efficacy of dual blood purification in the treatment of refractory pediatric septic shock and found that the levels of lactate and inflammatory factors significantly decreased and that oxygen metabolism significantly improved in children after treatment, suggesting that dual blood purification is an effective method for refractory pediatric septic shock. These results provided a basis for the treatment of severe infections in the present study.

In most studies of the effects of CBP on the haemodynamics in severe sepsis, only one single blood purification mode was used. A literature review revealed that a few studies on this topic have been reported. Eid et al. (20) successfully treated a patient with septic shock caused by necrotising fasciitis by providing ECLS combined with integrated blood purification treatment (CVVHDF plus HP), and found that heart rate, systolic blood pressure, diastolic blood pressure, mean arterial pressure, central venous pressure and stroke output were significantly improved in the patient after the combined treatment, without significant effect on cardiac output. In practice, this integrated blood purification therapy can be integrated into the standard care protocols for pediatric patients with sepsis, particularly those who do not respond adequately to conventional treatments. By incorporating blood purification techniques, clinicians can offer a more personalized approach to managing sepsis, targeting the underlying pathophysiological disturbances that are not addressed by antimicrobial therapy alone.

Parameters such as diastolic blood pressure and mean arterial pressure did not show significant changes after treatment in this study, which may seem contradictory. This variation can be attributed to the complex nature of sepsis and the individualized response to treatment. Diastolic blood pressure and mean arterial pressure are influenced by multiple factors, including vascular resistance and the patient’s baseline cardiac function. The lack of significant improvement in these parameters may reflect the residual effects of septic shock on vascular tone, which blood purification alone may not fully address. Additionally, the concurrent use of vasoactive drugs could stabilize mean arterial pressure to a certain extent, potentially masking the effects of blood purification. These findings underscore that while integrated blood purification can enhance overall cardiovascular performance, its effects on specific haemodynamic parameters may vary. This variability highlights the need for comprehensive management strategies that combine blood purification with other therapeutic measures, such as careful fluid management and tailored use of vasoactive agents, to optimize patient outcomes.

This study has a number of limitations. First, the sample size was small and no appropriate control group was used. Therefore, large-scale prospective controlled studies are needed to further verify the efficacy of integrated blood purification in the treatment of children with severe sepsis. Second, oxygen inhalation and mechanical ventilation were provided to the patients following admission to maintain adequate oxygen supply, and the blood samples for blood gas analysis prior to blood purification therapy were collected after these active treatments, which might affect the oxygen delivery before and after treatment. While we intended to monitor Pv-aCO_2_ or Pv-aCO_2_/CavO_2_ to better reflect tissue hypoxia before and after blood purification, limitations in clinical equipment and technology prevented us from obtaining these measurements in pediatric patients with septic shock. Additionally, while nonparametric tests were used due to the small sample size and non-normal distribution of the data, the limited statistical power may have affected the reliability of the conclusions. Finally, the use of vasoactive drugs, rapid fluid rehydration, transfusion of blood products and other procedures affected the haemodynamic outcomes of the children, and these confounding factors were not easy to control.

In summary, integrated blood purification improves the haemodynamics and oxygen metabolism indicators in children with severe sepsis and can serve as an adjunctive treatment for severe infections in children. These results support the recommendation of integrated blood purification as part of a multimodal approach to sepsis treatment in pediatric intensive care units, emphasizing its role in improving patient outcomes through enhanced haemodynamic and metabolic control. Future research should focus on optimizing treatment protocols and exploring the long-term benefits of this approach in larger, controlled studies.

## Data Availability

The original contributions presented in this study are included in this article/supplementary material, further inquiries can be directed to the corresponding author.
